# Microvesicles Derived from Human Umbilical Cord Mesenchymal Stem Cells Facilitate Tubular Epithelial Cell Dedifferentiation and Growth via Hepatocyte Growth Factor Induction

**DOI:** 10.1371/journal.pone.0121534

**Published:** 2015-03-20

**Authors:** Guan-qun Ju, Jun Cheng, Liang Zhong, Shuai Wu, Xiang-yu Zou, Guang-yuan Zhang, Di Gu, Shuai Miao, Ying-jian Zhu, Jie Sun, Tao Du

**Affiliations:** 1 Department of Urology, Shanghai Children’s Medical Center, School of Medicine, Shanghai Jiao Tong University, Shanghai, China; 2 Department of Urology, Shanghai First People's Hospital, School of Medicine, Shanghai Jiao Tong University, Shanghai, China; 3 Department of Urology, Henan Provincial People’s Hospital, Zhengzhou, China; 4 Department of Urology, Nantong Tongzhou People’s Hospital, Nantong, China; 5 Department of Urology, Qingdao Municipal Hospital, Qingdao, China; Center for Molecular Biotechnology, ITALY

## Abstract

During acute kidney injury (AKI), tubular cell dedifferentiation initiates cell regeneration; hepatocyte growth factor (HGF) is involved in modulating cell dedifferentiation. Mesenchymal stem cell (MSC)-derived microvesicles (MVs) deliver RNA into injured tubular cells and alter their gene expression, thus regenerating these cells. We boldly speculated that MVs might induce HGF synthesis via RNA transfer, thereby facilitating tubular cell dedifferentiation and regeneration. In a rat model of unilateral AKI, the administration of MVs promoted kidney recovery. One of the mechanisms of action is the acceleration of tubular cell dedifferentiation and growth. Both in vivo and in vitro, rat HGF expression in damaged rat tubular cells was greatly enhanced by MV treatment. In addition, human HGF mRNA present in MVs was delivered into rat tubular cells and translated into the HGF protein as another mechanism of HGF induction. RNase treatment abrogated all MV effects. In the in vitro experimental setting, the conditioned medium of MV-treated injured tubular cells, which contains a higher concentration of HGF, strongly stimulated cell dedifferentiation and growth, as well as Erk1/2 signaling activation. Intriguingly, these effects were completely abrogated by either c-Met inhibitor or MEK inhibitor, suggesting that HGF induction is a crucial contributor to the acceleration of cell dedifferentiation and growth. All these findings indicate that MV-induced HGF synthesis in damaged tubular cells via RNA transfer facilitates cell dedifferentiation and growth, which are important regenerative mechanisms.

## Introduction

AKI is considered a robust predictor of progression to chronic kidney disease and a major contributor to chronic renal failure [1–3]. Complete repair during AKI leaves no lasting evidence of damage, whereas aberrant and inadequate repair during AKI results in the formation of fibrotic lesions [1, 4, 5]. Therefore, the recovery phase of AKI may represent the best opportunity to reverse the harmful outcomes of AKI [6]. The development of new strategies to facilitate tissue repair during acute injury events is urgently needed and warranted for halting the ensuing fibrosis.

The pathophysiological process of AKI involves acute inflammation and injury to the tubular epithelium, followed by a repair process that restores epithelial integrity and function [7]. The contributions of the tubular epithelium to the pathophysiology of ischemic AKI are protean. The epithelium is not merely a passive victim of injury but is the principal participant in the kidney repair process [5, 7]. Researchers have determined that regeneration by surviving tubular cells is the predominant repair mechanism after ischemic AKI [8]. The surviving epithelial cells dedifferentiate and proliferate to replace the dying cells without a source of distinct progenitor cells [9].

In fact, epithelial dedifferentiation is an integral part of the repair process that, if correctly regulated, promotes cell survival, migration and proliferation, providing the building blocks for tubule regeneration [8]. Some reparative or survival growth factors synthesized in tubular cells, including HGF, insulin-like growth factor-1 (IGF-1), transforming growth factor-β1 (TGF-β1) and epidermal growth factor (EGF), exert paracrine effects to promote cell dedifferentiation and regeneration via cell-cell crosstalk mechanisms [8, 10]. Therefore, the induction of growth factor synthesis in the tubular epithelium may be favorable for cell dedifferentiation, survival and proliferation.

MSCs alleviate AKI-induced inflammation and accelerate kidney recovery in a paracrine/endocrine manner [11, 12]. Intriguingly, the efficacy of MSC-derived MVs for kidney repair following AKI is similar to that of cells [13–17], which indicates that MVs are critical mediators. MVs, which shuttle selected patterns of RNA, are regarded as vehicles for genetic information exchange between cells [18, 19]. Recently, MVs from MSCs have been shown to deliver mRNA, regulatory micro-RNA and transcriptional factors to injured tissue cells, thus leading to alteration of cell phenotype and function [19–21]. In our recent study, MVs derived from human umbilical cord MSCs (hUC-MSCs) promote human renal cancer cell proliferation and aggressiveness by inducing HGF synthesis [22]. The pro-tumor effects of MVs are attributable to RNA transfer [22]. MVs may induce HGF expression in damaged tubular cells via RNA transfer, thereby accelerating cell dedifferentiation and regeneration.

In a rat model of ischemic AKI, hUC-MSC-derived MVs accelerated kidney recovery and retarded fibrogenesis, and facilitating tubular cell dedifferentiation and proliferation was one of the mechanisms of action. MVs administration induced native (rat) and foreign (human) HGF synthesis in damaged rat tubular cells. RNase treatment inhibited the effects of MVs, highlighting the pivotal role of RNA transfer by MVs. We further demonstrated that HGF induction is a crucial contributor to the acceleration of tubular cell dedifferentiation and growth. Therefore, enhancing HGF synthesis via RNA transfer facilitates tubular cell dedifferentiation and proliferation and is a novel regenerative mechanism of MSC-derived MVs.

## Materials and Methods

### Ethics Statement

In this study, all research involving human participants was approved by the institutional review board of the Chinese Academy of Medical Science and Medical School of Shanghai Jiao Tong University. Human individuals in this study provided written informed consent to participate in this research. This study was performed in strict accordance with the recommendations outlined in the Guide for the Care and Use of Laboratory Animals of Shanghai Jiao Tong University. The protocol was approved by the Committee on the Ethics of Animal Experiments of Shanghai Jiao Tong University. All surgery was performed under sodium pentobarbital anesthesia, and all efforts were made to minimize suffering.

### Isolation and Characterization of hUC-MSCs and of MVs

hUC-MSCs were isolated and characterized as described previously [23]. The cells at the 3rd to 6th passage were used in in vitro and in vivo experiments. MVs released by hUC-MSCs were isolated and characterized as described previously [24]. For the preparation of MVs, hUC-MSCs were cultured in low-glucose DMEM deprived of FBS and supplemented with 0.5% bovine serum albumin (BSA) (Sigma-Aldrich, St. Louis, MO, USA) overnight. The supernatants were collected and centrifuged at 2000g for 20 min to remove debris. The cell-free supernatants were ultra-centrifuged at 1×10^5^g (Beckman Coulter Optima L-80K ultracentrifuge; Fullerton, CA, USA) for 1h at 4℃. The supernatants were abandoned and the isolated MVs were suspended with M199 (Sigma-Aldrich) containing 25mM HEPES (PH 7.4) and submitted to a second ultracentrifugation under the same conditions. MVs were re-suspended in serum-free M199. The size of the isolated MVs ranged from 80 nm to 1000 nm, with a mean value of 142 nm. Flow cytometric analyses of MVs indicated the presence of CD9, CD29, CD44, CD63, CD73 and CD105, but not CD34 and CD45. The protein content of MVs was quantified using the Bradford method, whereas a limulus test was employed to exclude endotoxin contamination of MVs. A portion of MVs was treated with 100 μg/mL RNase (Fermentas, Burlington, ON, Canada) for 3 h at 37°C, and the reaction was stopped by the addition of RNase inhibitor (Fermentas). After ultracentrifugation at 1×10^5^g (Beckman Coulter Optima L-80K ultracentrifuge; Fullerton, CA, USA) for 1 h at 4°C, the MVs were suspended in M199 (RNase-MVs). The spectrophotometer analysis revealed that most of the RNA in the MVs, which was extracted using Trizol reagent (Invitrogen, Carlsbad, CA, USA), was degraded by the RNase treatment (MVs: 1.2 ± 0.2 μg RNA/mg protein; RNase-MVs: less than 0.17 μg RNA/mg protein).

### Animal Model of AKI

Adult male Sprague-Dawley (SD) rats weighing approximately 180 to 210 g were used. The rats were housed at a constant temperature and humidity, with a 12:12-h light-dark cycle, and a standard diet and water were freely accessible. The rats were housed in a ventilated cage system.

To establish unilateral ischemic AKI, the blood flow to the left kidney was blocked for 60 min. Sham-operated animals underwent a sham operation. Immediately after reperfusion, MVs (30 μg), RNase-MVs in 0.5 ml M199 (vehicle), or vehicle were injected into AKI animals via the vena caudalis. The animals were randomized according to the various treatments: 1) Sham animals (n = 24); 2) AKI plus MV-injected animals (n = 24); 3) AKI plus RNase-MV-injected animals (n = 24); and 4) AKI plus vehicle-injected animals (n = 24). The animals were sacrificed at 24 h, 48 h, 1 wk or 2 wk post-treatment. Kidney samples were collected at the time of sacrifice and submitted for the appropriate testing.

### Renal Function

Serum creatinine was measured using a colorimetric micro-plate assay based on the Jaffe reaction (Bio-Assay Systems, Hayward, CA, USA). A colorimetric assay kit was used to quantify the levels of blood urea nitrogen (BUN) according to the manufacturer’s protocol (Bio-Assay Systems). These measurements, as well as enzyme-linked immunosorbent assay (ELISA) analysis and tissue collagen assays, were performed by several independent laboratory workers blinded to the origin of the samples.

### Masson’s Trichrome Staining

Masson’s trichrome staining was employed to assess collagen deposition in the kidney tissues. The fraction of renal tissues replaced by collagen was quantitatively determined using a point-counting technique in consecutive microscopic fields (×100 magnification) for each rat (n = 6 rats, each group) under a 176-point grid. All of the sections were reviewed by a blinded doctor.

### Immunohistochemistry Staining

Briefly, the slides were incubated with mouse anti-PCNA (dilution: 1:500; Abcam, Cambridge, UK), anti-α-smooth muscle actin (α-SMA) (dilution: 1:500; Abcam, Cambridge, UK), anti-vimentin antibody (dilution: 1:250; Abcam, Cambridge, UK), rabbit anti-HGF antibody (dilution: 1:200; Abcam, Cambridge, UK) or anti-human HGF antibody (dilution: 1:250; Abcam, Cambridge, UK), followed by a HPR-conjugated secondary antibody, using DAB as the substrate. The negative controls were performed by replacing the primary antibodies with non-immune immunoglobulins of the same iso-type. The nuclei were counterstained with Harris hematoxylin. Tubular cell apoptosis was assessed using a terminal transferase-mediated dUTP nick-end labeling (TUNEL) assay using an in situ cell death detection kit (Roche, Mannheim, Germany).

### Real-time Reverse Transcription (RT)-PCR

Total RNA was isolated from kidney samples or from cell lysates using TRIZOL Reagent (Invitrogen, Carlsbad, CA, USA) according to the manufacturer’s protocol. Total RNA was reverse transcribed using a Moloney murine leukemia virus (M-MLV) reverse transcriptase kit (Promega, Madison, WI, USA) and oligo(dT) primers (Invitrogen, Carlsbad, CA, USA) for 60 min at 42°C. Real-time PCR was performed by TaqMan gene expression assays (Applied Bio-Systems, Foster City, CA, USA) for the detection of mRNA expression. Real-time PCR was performed using the following primers:

TGF-β1: 5′-gaaggacctgggttggaagt-3′, 3′-gagatgttggttgtgttgggc-5′;

IGF-1: 5′-ctcctagtccctgcctctta-3′, 3′-tatccaccaacttaccaa-5′;

EGF: 5′-catctgtccagcaggttcagt-3′, 3′-cactaacgaaaggacccatgc-5′;

Rat HGF: 5′-cctatttcccgttgtgaag-3′, 3′-gtcatcccacctaccaatca-5′;

Rat β-actin: 5′-cctctatgccaacacagt-3′, 3′-gacacacctaaccaccga-5′;

Human HGF: 5′-CTCTGGTTCCCCTTCAATAG-3′, 3′GATAGCCCCATTTCTGGATGTC-5′;

Human β-actin: 5′-AAGGTGACAGCAGTCGGTT-3′, 3′GGAGAGGGTTCAGGTGTGT-5′;

The quantification of the target gene was normalized by β-actin, which is an internal control gene. The Ct (threshold cycle) for the target gene and for β-actin was determined for each sample. The values were expressed relative to a reference sample (samples from sham-operated rat kidney tissue or rat tubular cells treated with vehicle). The experimental samples were expressed as an n-fold difference relative to the reference sample. The relative mRNA expression was calculated using the 2^−ΔΔCT^ method. Triplicate reactions were performed for each sample.

### Western Blot

Protein extracts (30 μg or 50 μg per lane) were electrophoresed and then transferred to polyvinylidene fluoride membranes. Immuno-blotting was performed by incubating each membrane with anti-HGF (dilution: 1:1000; Abcam, Cambridge, UK), anti-proliferating cell nuclear antigen (PCNA) (dilution: 1:1000; Abcam, Cambridge, UK), anti-vimentin (dilution: 1:1000; Abcam, Cambridge, UK), anti-p-ERK1/2 (dilution: 1:1000; Abcam, Cambridge, UK), anti-glyceraldehyde-3-phosphate dehydrogenase (GAPDH) or anti-β-actin primary antibody overnight at 4°C. After being washed in PBS, each membrane was incubated for 1 h with a secondary peroxidase-conjugated antibody at room temperature. The bands were developed using enhanced chemi-luminescence (Amersham Pharmacia Biotech, Piscataway, NJ, USA). The density of each band was determined, and each determination was repeated twice to confirm the reproducibility.

### In Vitro Tubular Cell Culture and Treatment

Tubular cells (1×10^5^) were cultured in epithelial cell medium (EPiCM) (ScienCell, San Diego, CA, USA) containing 2% fetal bovine serum (FBS) until the cells reached 80% confluence. The cells were cultivated in a humidified atmosphere containing 2% O_2_ and 5% CO_2_ at 37°C for 1 h (Incubator: Binder, Germany) and then incubated with MVs (30 μg), RNase-MVs or vehicle under an ambient oxygen concentration (21%). After 24 or 48 h of incubation, the cells or the supernatants were harvested, respectively. For the preparation of the conditioned medium (CM), the collected supernatants were centrifuged at 2000g for 20 min to remove debris and then frozen at -80°C until use. The cell number was estimated using a hemocytometer, whereas Trypan blue exclusion was employed to assess cell viability. The HGF level in the CM was measured using a rat HGF ELISA Kit (R&D Systems, Minneapolis, MN, USA). The ELISA results were normalized using the cell numbers in the culture. Total RNA and protein extracted from tubular cells was submitted to RT-PCR and western blot to determine HGF or human HGF mRNA and HGF protein expression levels. All samples were frozen at -80°C until analysis. All experiments were performed at least in triplicate. A portion of the cells incubated with MVs, RNase-MVs or vehicle for 24 or 48 h were fixed in 4% paraformaldehyde and submitted to HGF staining.

For identifying human HGF protein expression in rat tubular cells, the hypoxia/re-oxygenation-injured cells were incubated on chamber slides and then treated with MVs, RNase-MVs or vehicle for 24 or 48 h. Subsequently, the slides were fixed in 4% paraformaldehyde and permeabilized with HEPES-Triton-X 100 buffer (Sigma, St Louis, MO, USA). Rabbit anti-human HGF antibody (dilution: 1:200; Abcam, Cambridge, UK) was employed as the primary antibody. The primary antibody was replaced by a non-immune immunoglobulin of the same iso-type for the negative control. Harris hematoxylin was added for nuclear counter-staining. Human HGF expression in hUC-MSCs and in rat tubular cells treated with vehicle were regarded as positive and negative controls, respectively.

To assess the effect of the CM (containing HGF) obtained from tubular cells treated with or without MVs on cell proliferation and apoptosis, tubular cells were seeded at 1×10^5^ cells/well into 6-well plates. At approximately 70% confluence, the cells were subjected to hypoxia/re-oxygenation as described above. Then, the culture solutions were changed to fresh medium with CM addition or without CM addition (BLANK). At 24 h later, the cells were either collected for PCNA protein detection (western blot) or fixed for TUNEL staining. PF2341066 (Pfizer, 9 μM) (c-Met inhibitor) or U0126 (Sigma, 10 μM) (MEK inhibitor) was also added to the CM. After 24 h of incubation, total cell proteins were isolated for western blot analysis of p-Erk1/2, vimentin and PCNA protein levels.

### Statistical Analyses

The data are expressed as the means ± SD. The primary data were collected using Excel, and statistical analyses were performed using Prism software (Graph Pad, San Diego, USA). Analysis of variance (ANOVA) followed by the Student-Newman-Keuls post-test and t-tests were performed to evaluate the differences between the data means, as appropriate. A P value of <0.05 was considered statistically significant.

## Results

### MVs released by hUC-MSCs reverse the abnormal kidney structure and function elicited by ischemic AKI

**Fig 1:**
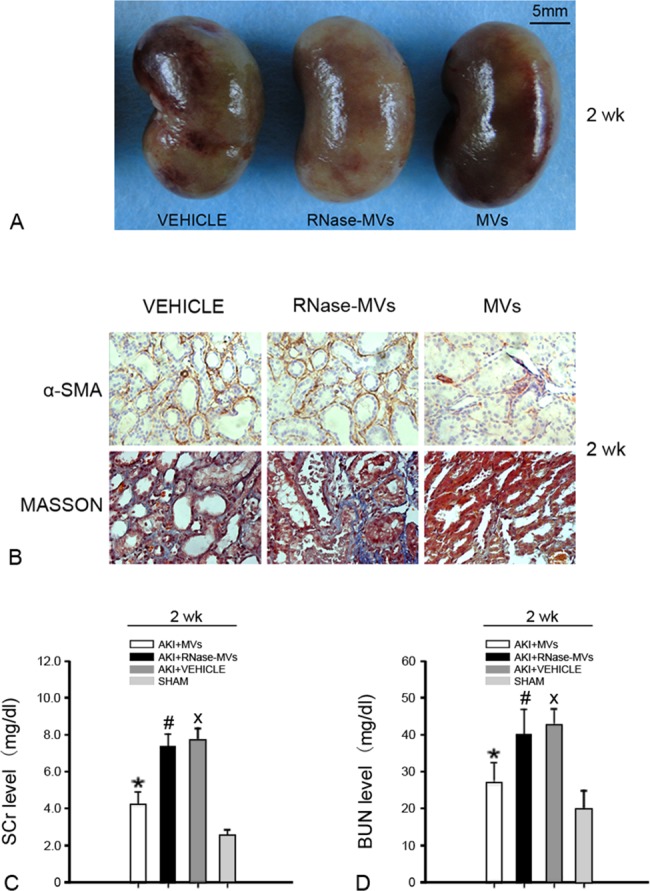
MV administration reverses the abnormal kidney structure and function elicited by AKI at 2 wk post-injury. (A) Representative micrographs of injured kidneys. In AKI animals treated with RNase-MVs or vehicle, the damaged kidneys display a mottled color, in contrast to a uniform color on the surfaces of the kidneys from MV-treated animals. (B) Representative micrographs illustrating α-SMA staining and Masson’s tri-chrome staining. Weaker positive staining was observed for α-SMA and for collagen on kidney sections from AKI animals receiving MV treatment compared with animals treated with RNase-MVs or vehicle. Magnification, ×40. (C) Serum creatinine value at 2 wk post-injury. Ischemic injury led to a significant increase in the serum creatinine level at 2 wk post-injury, which was inhibited by MV treatment. All quantitative data were obtained from 6 different animals for each experimental condition. *P<0.01, AKI+MVs vs. AKI+VEHICLE; #P<0.01, AKI+RNase-MVs vs. AKI+MVs; xP<0.001, AKI+VEHICLE vs. SHAM. (D) BUN value at 2 wk post-injury. MV administration also greatly inhibited the increase in the BUN level, which occurred at 2 wk post-injury. *P<0.05, AKI+MVs vs. AKI+VEHICLE; #P<0.05, AKI+RNase-MVs vs. AKI+MVs; xP<0.001, AKI+VEHICLE vs. SHAM.

At 2 wk post-injury, MV administration resulted in a marked reduction of collagen deposition in the affected kidney (P<0.01, Fig. 1B). Fewer α-SMA-positive kidney cells were found in MV-treated AKI animals (Fig. 1B). α-SMA expression is a key feature of myofibroblasts [25], which are the primary effector cells in the pathogenesis of fibrosis [26]. Therefore, this decline in myofibroblast accumulation accounts for the observed reduction in collagen deposition. Kidney dysfunction, which generally occurs at 2 wk post-injury, was also greatly ameliorated by MV administration (P<0.05, Fig. 1C and 1D). All of these effects of MV administration were abrogated by RNase treatment (P<0.05, Fig. 1B-1D).

### At the initial stage of AKI, MV administration accelerates tubular cell dedifferentiation and growth and inhibits apoptosis

**Fig 2:**
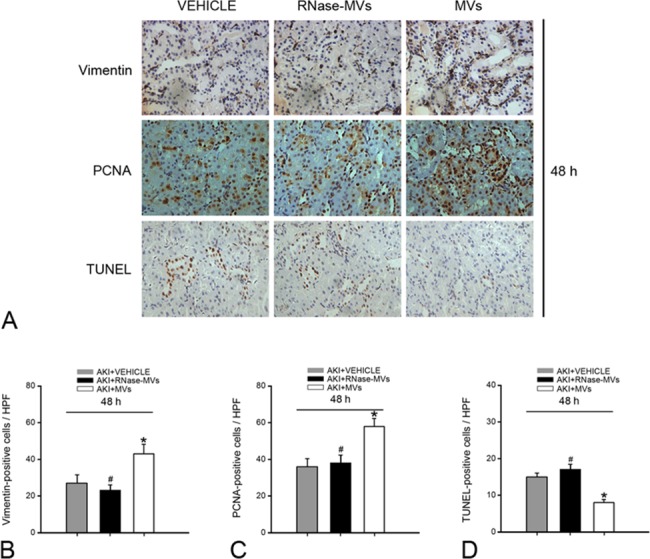
MV administration promotes tubular cell dedifferentiation and proliferation at 48 h post-injury, whereas cell apoptosis is inhibited. Representative micrographs showing vimentin, PCNA and TUNEL staining of tubular cells. Immuno-staining for vimentin and PCNA proteins, which are indictors for tubular cell dedifferentiation and cell proliferation, respectively, was employed. TUNEL staining was used to detect cell apoptosis. In contrast to the rats treated with vehicle or with RNase-MVs, the rats receiving MV treatment displayed more PCNA- and vimentin-positive stained tubular cells and fewer TUNEL-positive cells on kidney tissue sections. Magnification, ×40.

PCNA staining and vimentin staining in tubule cells were adopted as indicators for cell proliferation and dedifferentiation, respectively. At 48 h, MV administration greatly accelerated tubular cell dedifferentiation, in parallel with an increased number of proliferating tubular cells (P<0.05, Fig. 2). Additionally, tubular cell apoptosis was remarkably inhibited (P<0.05, Fig. 2). As expected, RNase pretreatment abolished these MV effects (P<0.05, Fig. 2). Tubular cell dedifferentiation is known to initiate cell growth and to protect cells against apoptosis [8], and our finding is consistent with this notion.

### Rat HGF expression in damaged rat tubular cells is substantially enhanced by MV administration

**Fig 3:**
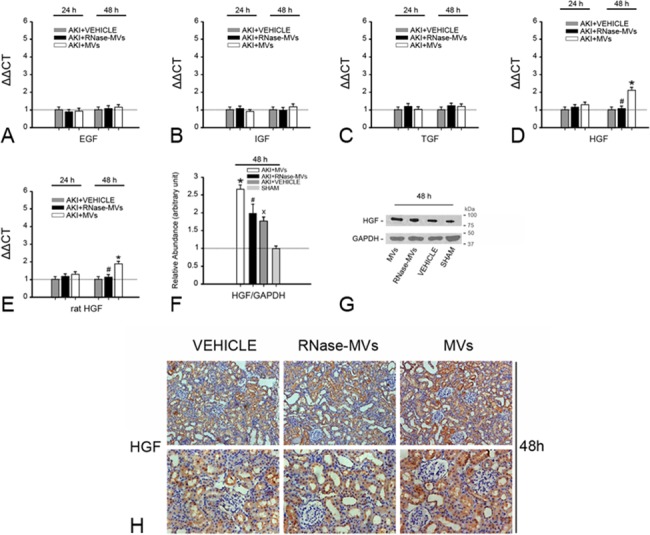
At 48 h post-injury, kidney HGF gene and protein expression is substantially enhanced by MV administration. (A)-(D) HGF gene expression in injured kidney tissues. MV administration led to a significant up-regulation of kidney HGF gene expression. The examination of rat HGF expression in kidney tissues using species-specific primers also indicated a similar result. As negative controls, no rat HGF mRNA was identified in MVs or in the cells of origin (hUC-MSCs). RNase pretreatment abolished the effect of MVs. By contrast, EGF, IGF-1 or TGFβ1 gene expression was not altered by MV administration. Gene expression levels in sham-treated samples were regarded as the baseline levels (dotted line). The relative expression levels of each gene were calculated using the 2−ΔΔCt method. The data were collected from 6 rats for each experimental condition. *P<0.05, AKI+MVs vs. AKI+VEHICLE; #P<0.05, AKI+RNase-MVs vs. AKI+MVs. (E) Densitometric analysis of kidney HGF protein expression. At 48 h, MV administration also resulted in a prominent increase in kidney HGF protein expression. This effect was abrogated by RNase pre-treatment. The values in the graph are expressed as densitometric ratios of HGF/GAPDH as fold changes compared with the control (sham-operated samples) (dotted line). *P<0.05, AKI+MVs vs. AKI+VEHICLE; #P<0.05, AKI+RNase-MVs vs. AKI+MVs; xP<0.01, AKI+VEHICLE vs. SHAM. (F) Representative gel photograph of kidney HGF protein expression. (G) HGF staining on kidney sections. Most of the positive staining was observed in damaged tubular cells. HGF staining of injured tubular cells was remarkably intensified in MV-treated animals at 48 h post-injury. Magnification, ×40.

Considering the role of growth factors produced by the tubular epithelium in tubular cell dedifferentiation and growth, we examined the effect of MVs on growth factor expression. In AKI animals, kidney HGF expression was greatly enhanced by MV administration at 48 h post-injury, as demonstrated by RT-PCR and by western blot analysis (P<0.05, Fig. 3D, 3F-G). Immunohistochemistry staining also revealed that a substantial intensification of HGF staining was observed in damaged tubular cells (P<0.05, Fig. 3H). By contrast, TGF-β1, IGF-1 or EGF expression was not significantly altered by MV administration (Fig. 3A-3C). Using species-specific (rat) primers, we also screened for rat HGF mRNA in damaged kidney tissues. We found that MV administration also markedly induced rat HGF expression (Fig. 3E). As a negative control, no rat HGF mRNA was detected in MVs or in their cells of origin (hUC-MSCs) (P<0.05, data not shown).

**Fig 4:**
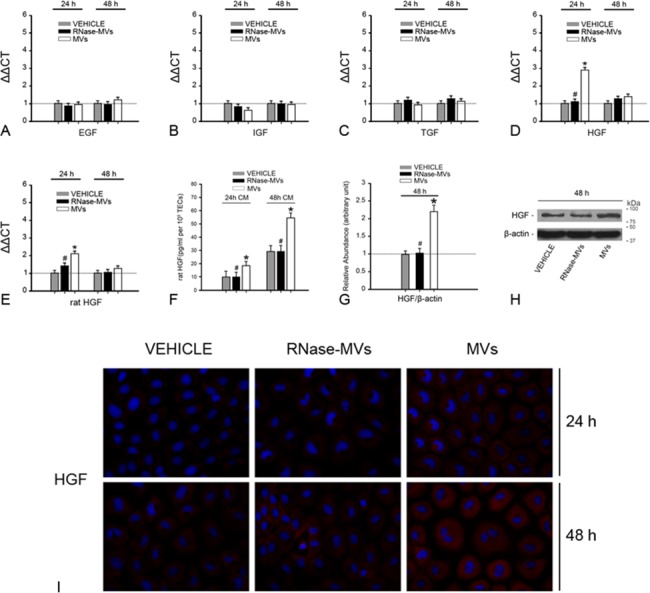
In the scenario of hypoxia/re-oxygenation, rat HGF expression in cultured tubular cells is significantly induced by MV administration. (A)-(E) HGF gene expression in damaged rat tubular cells. HGF gene expression in injured rat tubular cells was significantly enhanced by MV addition. The examination of rat HGF gene expression using species-specific primers (rat) also revealed a similar result. By contrast, EGF, IGF-1 or TGF-β1 gene expression remained unchanged by MV administration. The gene expression levels in the vehicle-treated cell samples were regarded as the baseline levels (dotted line). The relative expression levels of each gene were calculated using the 2−ΔΔCt method. The data were collected from 5 independent experiments. *P<0.05, MVs vs. VEHICLE; #P<0.05, RNase-MVs vs. MVs. (F) Rat HGF level in the conditioned medium (CM) of tubular cells. Compared with vehicle or with RNase-MVs, MVs caused a remarkable increase in the rat HGF level in the CM after 24 or 48 h of incubation with tubular cells. TECs: tubular epithelial cells. *P<0.02, MVs vs. VEHICLE; #P<0.05, RNase-MVs vs. MVs. (G) Densitometric analysis of HGF protein expression. At 48 h of incubation with tubular cells, MVs resulted in a marked increase in HGF protein expression. The values in the graph are expressed as densitometric ratios of HGF/β-actin as fold changes compared with the control (vehicle-treated cell samples) (dotted line). *P<0.01, MVs vs. VEHICLE; #P<0.01, RNase-MVs vs. MVs. (H) Representative gel photograph of HGF protein expression. (I) HGF staining in injured tubular cells. Stronger HGF-positive staining was observed in tubular cells incubated with MVs compared with RNase-MVs or vehicle, particularly after 48 h of incubation.

To verify the in vivo findings, some in vitro experiments were performed. In the scenario of hypoxia/re-oxygenation, HGF gene expression in rat tubular cells was markedly up-regulated after 24 h of incubation with MVs, whereas TGF-β1, IGF-1 or EGF gene expression remained unaffected (Fig. 4A-4D). Using species-specific primers (rat), we further demonstrated that rat HGF gene expression was significantly enhanced by MV addition (P<0.05, Fig. 4E). Moreover, stronger staining for HGF was observed in injured tubular cells treated with MVs (P<0.05, Fig. 4I), while western blot analysis revealed that there was more HGF protein expression in the cells (Fig. 4G-4H). The conditioned medium (CM) of MV-treated tubular cells also contained higher levels of rat HGF (P<0.05, Fig. 4F). All of these findings indicate that MVs greatly amplify the synthesis and release of native HGF by tubular cells. RNase pretreatment inhibited the in vivo and in vitro effects of MVs (P<0.05, Fig. 3–4).

### Human HGF mRNA originally residing in MVs enters the injured rat tubular cells and is translated into the HGF protein

**Fig 5:**
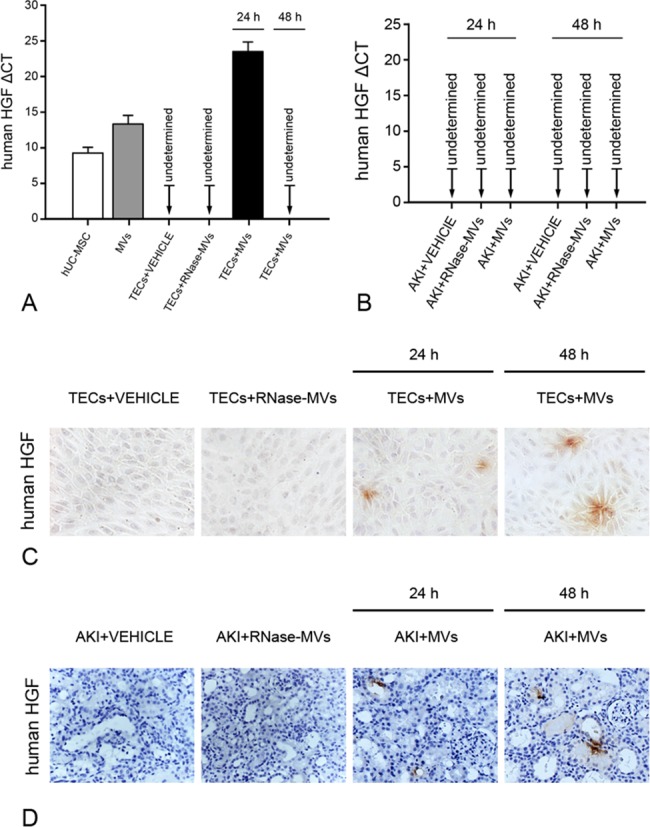
Either in vivo or in vitro, human HGF mRNA present in MVs enters injured rat tubular cells and is translated into the corresponding protein. (A) Human HGF gene expression in cultured rat tubular cells. The human HGF gene transcript was detectable in MVs and in the cells of origin. After 24 h of incubation with MVs, the human HGF mRNA was present in injured rat tubular cells, whereas human HGF mRNA was absent in cells incubated with vehicle or with RNase-MVs. The Ct for human HGF and β-actin (rat or human) was determined for each sample. The data were collected from 5 independent experiments. TECs: tubular epithelial cells. (B) Human HGF gene expression in rat kidney tissues. The human HGF mRNA was not detected in the affected kidney tissues of MV-treated AKI animals at any given points in time. The Ct for human HGF and rat β-actin was determined for each kidney sample. The data were collected from 3 samples for each group. (C) Human HGF in vitro staining. After MV exposure for 24 or 48 h, a few injured rat tubular cells displayed human HGF-positive expression in the cytoplasm. As a control, no positive staining was observed in cells exposed to vehicle or RNase-MVs. Magnification, ×20. (D) Human HGF in vivo staining. At 24 or 48 h following MV administration, the human HGF protein was detected in a few tubular cells. No positive-stained kidney cells were identifiable in animals treated with vehicle or with RNase-MVs. Magnification, ×40. AKI: acute kidney injury.

After 24 or 48 h of incubation with MVs, immunocytochemistry staining detected the human HGF protein in a few rat tubular cells (Fig. 5C). Using species-specific primers (human), real-time PCR examination also indicated the presence of human HGF mRNA in MV-incubated rat tubular cells (24 h), as well as in MVs and in their cells of origin (hUC-MSCs) (Fig. 5A). By contrast, in the cells incubated with vehicle or RNase-MVs, neither the human HGF protein nor its gene transcript was detected at any given point in time (Fig. 5A and 5C). In vivo, the human HGF protein was also unambiguously identified in tubular cells of MV-treated AKI animals (Fig. 5D). As controls, no positive-stained cells were detectable in AKI animals receiving vehicle or RNase treatment (Fig. 5D). At any given point in time, we did not detect human HGF mRNA in kidney tissue samples (Fig. 5B). These findings indicate that human HGF mRNA existing in MVs enters rat tubular cells and then is translated into the corresponding protein, which is another mechanism of HGF induction (induction of foreign HGF synthesis).

### The CM of MV-treated injured tubular cells exhibits stronger pro-proliferative and anti-apoptotic effects

**Fig 6:**
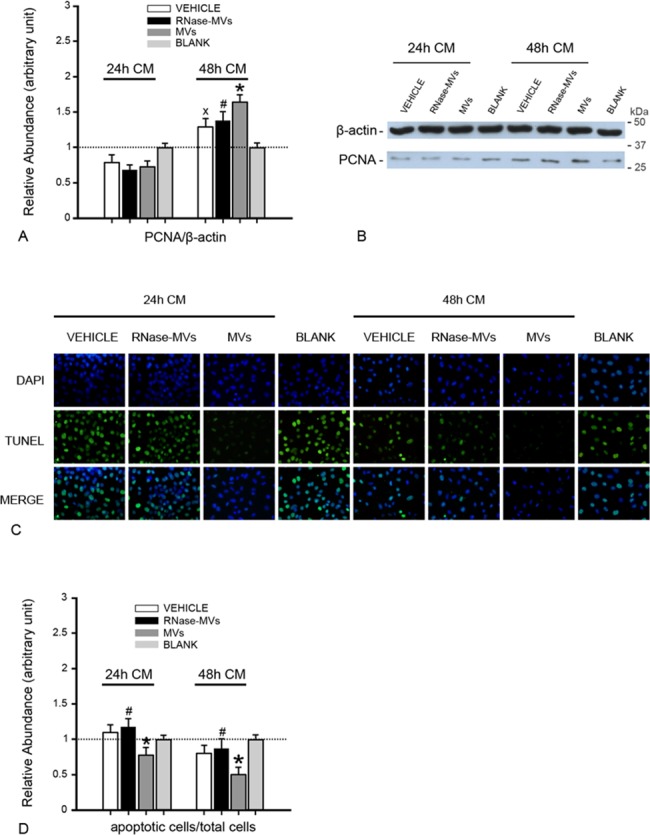
The CM of MV-treated injured tubular cells promotes cell proliferation and inhibits apoptosis. (A) Densitometric analysis of PCNA protein in tubular cells. The CM of injured tubular cells treated with MVs for 48 h elicited a marked increase in PCNA protein expression in tubular cells. The values in the graph are expressed as densitometric ratios of PCNA/β-actin as fold changes compared with the control (BLANK) (dotted line). CM: conditioned medium of tubular cells; BLANK: medium without exposure to injured tubular cells. *P<0.01, MVs vs. VEHICLE; #P<0.05, RNase-MVs vs. MVs; xP<0.05, VEHICLE vs. BLANK. (B) Representative gel photograph of PCNA protein expression. (C) TUNEL staining. In the presence of the CM of MV-treated tubular cells, few TUNEL-positive cells were observed under ×20 magnification. (D) Quantitative evaluation of cell apoptosis. Cell apoptosis was substantially inhibited by the CM of MV-treated tubular cells. The values in the graph are expressed as ratios of apoptotic cells/total cells as fold changes compared with the control (BLANK) (dotted line). *P<0.01, MVs vs. VEHICLE; #P<0.05, RNase-MVs vs. MVs.

The conditioned medium (CM) of tubular cells represents the cellular response to acute hypoxia injury. To confirm the effects of the CM (containing HGF) on tubular cell proliferation and apoptosis, the CM (24 or 48 h) was added to the medium of hypoxia/re-oxygenation-subjected tubular cells. In contrast to the BLANK, the CM (48 h) of vehicle-treated tubular cells vehicle significantly accelerated tubular cell growth (P<0.05, Fig. 6A and 6B), indicating that tubular cells do release some bioactive factors (including HGF) in response to damage, thereby facilitating cell regeneration. Interestingly, the CM (48 h) obtained from MV-treated cells exhibited a greater pro-proliferative effect (P<0.05, Fig. 6A and 6B). Additionally, tubular cell apoptosis was also strongly inhibited (P<0.05, Fig. 6C and 6D).

### The effects of the MV-treated damaged tubular cell CM are completely abrogated by a c-MET inhibitor or MEK inhibitor

**Fig 7:**
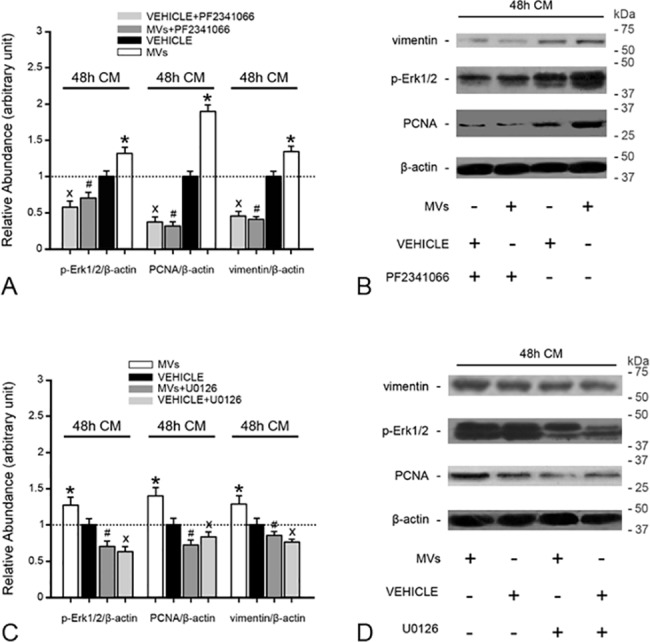
c-Met inhibitor or MEK inhibitor addition abrogates tubular cell dedifferentiation and growth, as well as Erk1/2 signaling activation induced by the CM of MV-treated injured tubular cells. (A), (C) Densitometric analysis of vimentin, PCNA and p-Erk1/2 protein levels in tubular cells. c-Met inhibitor or MEK inhibitor addition completely abrogated the up-regulation of vimentin, PCNA and p-Erk1/2 protein expression induced by CM of damaged tubular cells treated with MVs. Values in the graph are expressed as densitometric ratios of vimentin/β-actin, PCNA/β-actin or p-Erk1/2/β-actin as fold changes compared with the control (CM of tubular cells treated with vehicle) (dotted line). *P<0.05, MVs vs. VEHICLE; #P<0.05, MVs+PF2341066 or U0126 vs. MVs; xP<0.05, VEHICLE+PF2341066 or U0126 vs. VEHICLE. (B), (D) Representative gel photograph of vimentin, PCNA and p-Erk1/2 protein expression.

The vimentin and PCNA proteins were considered markers of cell dedifferentiation and growth, respectively, in tubular cells. We found that the CM of MV-treated tubular cells remarkably promoted cell dedifferentiation and growth, as well as Erk1/2 signaling activation (P<0.05, Fig. 7A-7D). Then, a c-MET inhibitor (PF2341066) was added to block the HGF/c-Met signaling pathway of tubular cells. Interestingly, PF2341066 completely abrogated Erk1/2 signaling activation, as well as the cell dedifferentiation and growth induced by the CM of MV-treated tubular cells (P<0.05, Fig. 7A and 7B). Furthermore, an Erk1/2 signaling inhibitor (U0126) exerted a similar effect (P<0.05, Fig. 7C and 7D). HGF can promote tubular cell dedifferentiation and migration via Erk1/2 signaling activation [8]; therefore, we believe that MV-induced HGF synthesis is an important contributor to the acceleration of tubular cell dedifferentiation and growth.

## Discussion

MSCs can protect and reverse ischemia- or toxic agent-elicited AKI. In some studies, a few cells were observed transiently residing in tubules after delivery, which suggests that MSCs exert a trophic effect by a paracrine action [27, 28]. In other studies, MSCs mitigate acute damage with no required cell presence in the kidney [23, 29], which indicates an endocrine mechanism of action. Therefore, the factor produced and secreted by MSCs promotes self-repair from cells surviving injury. In this context, MVs released from MSCs may be pivotal mediators because these MVs may induce phenotypic and functional changes in the recipient cells via a variety of complicated mechanisms [19, 20]. Recently, MVs were found to mimic the efficacy of MSCs on the recovery from ischemic or toxic AKI [13, 14].

In a rat model of unilateral ischemic AKI, we demonstrated that MVs released from hUC-MSCs could ameliorate kidney function and retard the development of renal fibrosis when administered at the beginning of kidney functional and morphologic alterations, which is consistent with previous reports. Considering that aberrant tissue repair is a trigger of fibrogenesis, the amelioration of acute injury repair may be a vital mechanism of action. Indeed, at 48 h post-injury, MVs triggered a strong proliferative response and anti-apoptotic events.

Replacing the lost tubular cells following episodes of acute injury is primarily due to the proliferation of surviving tubule cells [9]. Histologic studies have revealed that cell loss during AKI is followed by the phenotypic dedifferentiation of surviving cells [30]. These partially dedifferentiated tubular cells undergo proliferation, thus providing the majority of cells that repopulate the tubule [31, 32]. Cell dedifferentiation also prevents injured cells from apoptosis, thus providing a chance for survival [33]. In the present study, the administration of MVs remarkably promoted tubular cell dedifferentiation at 48 h post-injury; this dedifferentiation is a key mechanism underlying the pro-proliferative and anti-apoptotic effects of MVs.

In the present study, we provided further insight into the mechanism by which MVs can induce tubular cell dedifferentiation. Given the destructive effect of RNase treatment, RNA capsulated by MVs may be a key. MVs, which are a novel avenue for cell-to-cell communication, can deliver mRNA and microRNA to targeted cells, thus mediating cellular gene expression [34, 35]. Once intravenously injected, MVs are primarily captured by damaged tubular cells during toxic AKI [14]. In a recent study, we also found that MVs derived from hUC-MSCs, which were delivered via the tail vein following ischemic AKI, primarily accumulated in the affected kidney [36]. Hence, MVs may alter tubular cell gene expression via RNA transfer.

Tubular cell dedifferentiation has been linked to an increase in the synthesis of some growth factors, such as IGF-1 [37], HGF [38] and EGF [39], by tubular cells. In the present study, the kidney HGF gene and protein expression levels were highly up-regulated, whereas increased HGF protein synthesis was observed in damaged tubular cells, together with prominent cell dedifferentiation and proliferation. By contrast, IGF-1, TGF-β1 and EGF expression was not affected by MV administration. Therefore, MVs seem to establish a milieu favorable for tubular cell dedifferentiation and regeneration through HGF induction.

Some in vitro experiments were performed to further corroborate this possibility. We found that MVs greatly induce rat HGF gene and protein expression in hypoxia/reoxygenation-subjected tubular cells. Moreover, the rat HGF protein was detectable in the conditioned medium (CM) of MV-exposed damaged tubular cells. These findings indicate that MVs induce native (rat) HGF synthesis in injured tubular cells similar to that observed in vivo. In addition, the in vivo and in vitro evidence indicates that human HGF mRNA present in MVs is delivered into damaged rat tubular cells and translated into the corresponding protein. Researchers have recognized that a horizontal transfer of mRNA mediated by MVs results in protein synthesis in target cells. Our study provides new evidence for this mechanism. We believe that this mechanism is another HGF induction mechanism. However, notably, only a few damaged tubular cells expressing the human HGF protein were observed in the in vivo and in vitro experimental settings. In our opinion, the induction of native HGF synthesis may be the mainstay of the inductive mechanisms.

Further experiments were performed to confirm that HGF induction is an important contributor to tubular cell dedifferentiation and growth. The conditioned medium (CM) of tubular cells represents the cellular response to acute insult. We evaluated the effect of the CM on tubular cell dedifferentiation and growth. Interestingly, the CM of MV-treated tubular cells (containing higher levels of HGF) not only strongly promoted cell dedifferentiation and growth but also led to remarkable Erk1/2 signaling activation. More importantly, these effects were completely abolished by a c-Met inhibitor or MEK inhibitor, indicating that MVs at least partially promote damaged tubular cell dedifferentiation and growth via inducing HGF synthesis.

Some concerns over the use of MSCs, such as a loss of control over time, vascular occlusion and mal-differentiation, as reviewed by Luigi Biancone et al. [40], have caused researchers to consider MVs released by MSCs. In the present study, we demonstrated that hUC-MSC-derived MVs ameliorate aberrant injury repair by facilitating tubular cell dedifferentiation and growth via RNA transfer. The induction of HGF synthesis in damaged tubular cells is an important contributor to this process. Over a long period, several bottlenecks have always occurred in translating the experimental results of HGF synthesis into a clinical benefit. One issue originates from the rapid clearance of HGF from the circulation, which makes sustaining a sufficient level in vivo difficult. MVs, which induce HGF synthesis, might circumvent this issue. The in situ production of HGF seems far more effective than simply injecting exogenous HGF. Due to HGF induction occurring in damaged tubular cells, the synthesized and released HGF could accelerate cell dedifferentiation and regeneration in a paracrine or autocrine manner. Further research should focus on the essence of RNA-mediated HGF expression to pave the way for the rational assembly of the RNA content of MVs.
